# Dihydroauroglaucin Isolated from the Mediterranean Sponge *Grantia compressa* Endophyte Marine Fungus *Eurotium chevalieri* Inhibits Migration of Human Neuroblastoma Cells

**DOI:** 10.3390/pharmaceutics14030616

**Published:** 2022-03-11

**Authors:** Marzia Vasarri, Giovanni Andrea Vitale, Giovanna Cristina Varese, Emanuela Barletta, Maria Valeria D’Auria, Donatella de Pascale, Donatella Degl’Innocenti

**Affiliations:** 1 Department of Experimental and Clinical Biomedical Sciences, University of Florence, Viale Morgagni 50, 50134 Florence, Italy; marzia.vasarri@unifi.it (M.V.); emanuela.barletta@unifi.it (E.B.); donatella.deglinnocenti@unifi.it (D.D.); 2 Department of Eco-Sustainable Marine Biotechnology, Stazione Zoologica Anton Dohrn, Villa Comunale, 80125 Naples, Italy; giovanniandrea.vitale@szn.it; 3 Department of Life Sciences and Systems Biology, Mycotheca Universitatis Taurinensis, University of Turin, Viale Mattioli 25, 10125 Turin, Italy; 4 Department of Pharmacy, University of Naples “Federico II”, 80131 Naples, Italy; madauria@unina.it

**Keywords:** *Eurotium chevalieri*, marine fungi, natural products, cell migration

## Abstract

Cancer cell migration is a hallmark of the aggressiveness and progression of malignancies such as high-risk neuroblastoma. Given the lack of effective therapeutic solutions to counteract cancer progression, basic research aims to identify novel bioactive molecules with inhibitory potential on cancer cell migration. In this context, this work investigated the role of members of the salicylaldehyde secondary metabolite set from the sponge endophyte fungus *Eurotium chevalieri* MUT 2316 as potential inhibitors of human neuroblastoma SH-SY5Y cell migration. Since tetrahydroauroglaucin (TAG) and dihydroauroglaucin (DAG) were isolated in large amounts, both were evaluated for their anticancer properties towards SH-SY5Y cells. Both molecules were found to be non-cytotoxic by MTT assay and cytofluorimetric analysis. Moreover, DAG showed efficacy in inhibiting the highly migratory phenotype of SH-SY5Y cells by wound healing assay; whereas TAG, although structurally similar to DAG, showed no anti-migratory effect. Therefore, this work provides good reasons to conduct further in vitro and in vivo studies focusing on DAG as a potentially useful migrastatic natural marine molecule.

## 1. Introduction

Endophytic marine fungi, especially those associated with sessile macroorganisms such as sponges [1], are promising sources of bioactive secondary metabolites. In the frame of our ongoing work on the valorisation of the biotechnological potential of marine fungi [2], we have recently reported the fungus *Eurotium chevalieri* MUT 2316, isolated from the Mediterranean sponge *Grantia compressa*, as a high-yield source of bioactive metabolites [3,4].

The strain was grown under 12 cultural conditions using the OSMAC (one strain many compounds) approach to expand the spectrum of secondary metabolites produced by the fungus according to the modulation of culture conditions [5,6]; yielding 10 compounds which were isolated and characterised. The identified compounds belong to three different classes of metabolites: anthraquinones, diketopiperazines, and prenylated benzaldehyde derivatives. In assessing bioactivity, members of this latter group dihydroauroglaucin (DAG), flavoglaucin (FG), and isodihydroauroglaucin (IDAG) were found to possess interesting activities, with DAG being able to completely inhibit influenza (IAV) viral replication. IDAG being active (minimum inhibitory concentration values ranging from 4 to 64 mg/mL) against reference strains and multidrug-resistant isolates of *Staphylococcus aureus* and *Streptococcus pneumoniae* [4], and DAG and FG showing a broad spectrum of inhibitory activity of the adhesion and/or growth of microfouler marine bacteria and microalgae [3].

Since the first report in 1934 of FG and DAG from the terrestrial fungus *Aspergillus glaucus* [7], this growing class of C7 alkylated salicylaldehyde derivatives [8] has been described to possess a broad spectrum of biological activities: antioxidant and radical scavenging properties [9,10,11], antimicrobial, antibiofilm [12], and antifouling activities [3], anti-inflammatory [13,14] and skin-protective properties under oxidative stress condition [15] as well as binding affinity to human opioid or cannabinoid receptors [16].

Marine natural products with a wide range of biological activities have long been recognised as a potential resource for drug design and treatment of a variety of human diseases, including cancer [17]. A hallmark of cancer aggressiveness is cancer cell migration, a critical driver of cancer progression to intractable metastatic disease [18]. Therefore, cell migration is an important target to curb the spread of cancer cells. Nevertheless, most current therapies are only designed [19] to kill proliferating cancer cells or to stop active oncogenic signalling. These standard therapies, while initially effective against cancer growth, have limited or no efficacy against metastasis [20].

The identification of naturally occurring migrastatic natural products, that have the potential to inhibit cancer cell migration is another important strategy for highly metastatic cancers without effective therapeutic options [21], such as high-risk neuroblastoma (NB).

NB accounts for approximately 10% of all solid tumours and occurs in very young children, with an average age of 17 months at diagnosis. In clinical practise, various anticancer drugs and therapies are used to prevent the high NB proliferation [22], but high-risk NB has a poor prognosis with an extremely poor overall survival rate [23].

The discovery of molecules that are effective against cancer cell migration and do not exhibit cytotoxicity is an important goal for the development of new approaches that reduce the toxicity and adverse effects of conventional drugs.

In this context, the aim of this study was to investigate the potential role of members of the salicylaldehyde set of secondary metabolites from the marine fungus *Eurotium chevalieri* MUT 2316 as potential inhibitors of cancer cell migration. For the optimised isolation of prenylated benzaldehyde derivatives, a cultivation of the strain *Eurotium chevalieri* MUT 2316 was subjected to an extraction and fractionation procedure slightly different from that previously reported. This procedure allowed the isolation of six metabolites, including high yields of tetrahydroauroglaucin (TAG), not isolated in the previous study [4], and dihydroauroglaucin (DAG). Since the prenylated benzaldehyde derivatives TAG and DAG were purified in high amounts in *Eurotium chevalieri* MUT 2316, they were selected for bioactivity investigation. Specifically, using SH-SY5Y human neuroblastoma cells as an in vitro cell model, the ability of TAG and DAG to inhibit the highly migratory behaviour of these cancer cells was investigated.

## 2. Materials and Methods

### 2.1. General Experimental Procedures

HRMS data were acquired in positive mode using a Thermo LTQ Orbitrap XL mass spectrometer (Thermo Fisher Scientific Inc., Waltham, MA, USA) linked to a Thermo U3000.

NMR spectra were recorded on Bruker Avance NEO 400 spectrometer equipped with a RT-DR-BF/1H-5 mm-OZ SmartProbe. Coupling constants (J) are given in Hertz (Hz), chemical shifts were given in δ (ppm) and were referred to the residual CHCl_3_ as the internal standard (δ_H_ = 7.26). Spin multiplicities are given as s (singlet), br s (broad singlet), d (doublet).

### 2.2. Reagents

Merck KGaA (Darmstadt, DA, Germany) provided Dulbecco’s modified Eagle’s medium (DMEM), foetal bovine serum (FBS), penicillin and streptomycin, L-glutamine, 1-(4,5-dimethylthiazol-2-yl)-3,5-diphenyl formazan (MTT), and all chemicals and solvents.

Bio-Rad (Hercules, CA, USA) provided the electrophoresis reagents. Table 1 shows the primary antibodies used with the specifications. Molecular Probes^TM^ (Invitrogen; Carlsbad, CA, USA) provided secondary antibodies as goat anti-rabbit and anti-mouse IgG HRP-linked. Sarstedt (Nümbrecht, Germany) provided plastics.

### 2.3. Eurotium chevalieri Cultivation

Ten agar plugs (obtained from the margin of an actively growing colony) of *Eurotium chevalieri* MUT 2316 were inoculated into 20 Erlenmeyer flasks (250 mL) containing 180 mL of Potato Dextrose Broth (PDB; potato extract 4 g, dextrose 20 g, agar 15 g, Sigma-Aldrich, Saint Louis, MO, USA—up to 1 L dH_2_O) supplemented with 3% NaCl. The flasks were incubated in the dark at 24 °C and 120 rpm for 14 days. The cultures were filtered to separate the fungal biomass from the broth.

### 2.4. Purification of Fungal Metabolites

Biomass was freeze-dried, suspended in a solution of EtOAc:CH_2_Cl_2_ (1:1, *v*/*v*), and homogenised using the Ultra Turrax. The sample was sonicated and filtered under vacuum to get rid of biomass. This step was repeated three times to increase the yield of the extracted metabolites.

The crude extract (370 mg) was further partitioned by dissolving and shaking with 1 L of CHCl_3_:H_2_O (1:1, *v*/*v*) using a separation funnel. After the two phases separated, the organic phase was collected and dried in a rotary evaporator to obtain 120 mg of extract.

The extract was dissolved in MeOH at a concentration of 25 mg/mL and processed using an UPLC equipped with a Fluka C-18 Brisa LC2 (5 μm, 4.6 mm i.d × 250 mm), as mobile phase A (H_2_O + 0.1% formic acid) and B (ACN + 0.1% formic acid) were used, flow rate was set at 1 mL/min, and separation was performed with the following gradient: the starting condition was set at 100% A, then it reached 100% B in 40 min, keeping these conditions for 8 min, finally it was re-equilibrated for the next run.

The abovementioned fractionation finally yielded six pure compounds: Compound **1** (1.2 mg, t_R_ 19.80), Compound **2** (0.6 mg, t_R_ 22.90), Compound **3** (1.2 mg, t_R_ 24.75), Compound **4** (13.5 mg, t_R_ 25.50), Compound **5** (17.1 mg, t_R_ 28.40), Compound **6** (2.1 mg, t_R_ 29.50). All the compounds except Compound **5** were isolated in our previous work [4].

Compound **1**: Erythroglaucin—red powder; pseudomolecular ion [M + H]^+^ at m/z 301.0703 (Δ = −1.211 ppm) compatible with the molecular formula C_16_H_13_O_6_.

Compound **2**: Physcion—orange powder; pseudomolecular ion [M + H]^+^ at m/z 285.0755 (Δ = −0.772 ppm) compatible with the molecular formula C_16_H_13_O_5_.

Compound **3**: Isodihydroauroglaucin—yellow powder; pseudomolecular ion [M + H]^+^ at m/z 301.1798 (Δ = −0.037 ppm) compatible with the molecular formula C_19_H_25_O_3_.

Compound **4**: Dihydroauroglaucin—yellow powder; pseudomolecular ion [M + H]^+^ at m/z 301.1796 (Δ = −0.767 ppm) compatible with the molecular formula C_19_H_25_O_3_.

Compound **5**: Tetrahydroauroglaucin—yellow powder; pseudomolecular ion [M + H]^+^ at m/z 303.1951 (Δ = −1.026 ppm) compatible with the molecular formula C_19_H_27_O_3_. ^1^H-NMR (CDCl_3_, 400 MHz) δ: 11.73 (1H, s, 2-OH), 10.13 (1H, s, 1-CHO), 7.04 (1H, s, H-4), 6.44 (1H, d, J = 16.2 Hz, H-1′), 5.95 (1H, m, H-2′), 5.29 (1H, bs, H-2″), 3.32 (2H, bs, H-1″), 2.40 (2H, bs, H-3′), 1.70 (3H, s, H-4″), 1.75 (3H, s, H-5″), 1.52 (2H, m, H-4′), 1.36 (4H, m, H-5′ and H-6′), 0.89 (3H, t, J = 5.8 Hz, H7′) (see Figure S1 in Supplementary Materials for ^1^HNMR spectrum). 

Compound **6**: Flavoglaucin—yellow powder; pseudomolecular ion [M + H]^+^ at m/z 305.2106 (Δ = −1.511 ppm) compatible with the molecular formula C_19_H_27_O_3_.

### 2.5. Cells and Culture Conditions

The American Type Culture Collection (ATCC^®^, Manassas, VA, USA) provided the human neuroblastoma SH-SY5Y cells. SH-SY5Y cells were grown at 37 °C in a humidified atmosphere of 5% CO_2_, in a mixture of 50% Ham’s F12 and 50% DMEM supplemented with L-glutamine (2 mM), streptomycin (100 mg/mL), penicillin (100 U/mL), and 10% FBS (complete medium).

At 80% confluence, SH-SY5Y cells were detached by trypsinisation (0.25% trypsin, 0.5 mM EDTA solution) and propagated after appropriate dilution.

Experiments were performed with cells treated with different concentrations of TAG or DAG (2.5–20 μM) in heat-inactivated FBS media (HI-FBS media). Cells treated with different doses of vehicle (i.e., DMSO) served as controls (Ctrl).

### 2.6. Evaluation of Cell Viability

The colorimetric MTT activity assay was used to investigate cell viability. Human neuroblastoma SH-SY5Y cells were seeded in a 96-well plate (5 × 10^3^ cells/well) overnight. Cells were then treated with TAG or DAG (2.5–20 μM) in HI-FBS medium for 24h. After washing cells with PBS, 100 μL/well of MTT solution (0.5 mg/mL) was added. Cells were lysed in DMSO (100 μL/well) after 1h of incubation at 37 °C in the dark and a wash in PBS. The iMARK microplate reader (Bio-Rad, Philadelphia, PA, USA) was used to measure absorbance at 595 nm. Data on relative cell viability were expressed as percentage of control cells. Cell viability was also evaluated by flow cytometer analysis using probe for identifying apoptotic cells, i.e., BV421 Annexin V (563973, BD Biosciences, Franklin Lakes, NJ, USA) and 7-Aminoactinomycin D (7-AAD) according to the manufacturer’s protocol. Briefly, SH-SY5Y cells were grown for 24h in the absence or presence of TAG or DAG (2.5–20 μM) in HI-FBS media. Cells (1 × 10^6^ cells/tube) were harvested, centrifuged, and resuspended in 1 mL of 1× Annexin Binding Buffer (100 mM 4-(2-hydroxyethyl)-1-piperazineethanesulfonic acid (HEPES), 140 mM sodium chloride, 25 mM calcium chloride, pH 7.4). Then, 100 µL of the cell suspension was mixed with 1 µL BV421 Annexin V and 5 µL of 7-AAD and incubated for 15 min at room temperature in the dark. Then, 400 μL of 1X Annexin Binding Buffer was added to each sample, and cells were analysed using BD FACS Canto II (BD Biosciences, Franklin Lakes, NJ, USA). Data were analysed using Diva software (BD Biosciences, Franklin Lakes, NJ, USA). Cellular distribution as a function of Annexin V and/or 7-AAD positivity allowed measurement of the percentage of viable cells (Annexin V and 7-AAD negative, Q4), early apoptosis (Annexin V positive and 7-AAD negative, Q3), or late apoptosis (Annexin V and 7-AAD positive, Q2). A minimum of 10,000 events was collected.

### 2.7. Detection of Autophagic Markers

SH-SY5Y cells (2 × 10^5^ cells) were seeded in 60 mm dishes in HI-FBS complete medium overnight. Cells were then treated with TAG or DAG at two different concentrations (2.5 and 5 μM), and after PBS washing, they were lysed at 24 h, in 80 μL of Laemmli buffer (62.5 mM Tris-HCl pH 6.8, 10% (*w*/*v*) SDS, 25% (*w*/*v*) glycerol) without bromophenol blue.

For each sample, 25 μg of protein was spiked with 0.5% (*v*/*v*) β-mercaptoethanol and bromophenol blue. Proteins were separated in 15% *w*/*v* PAGE and then transferred to PVDF membranes. Membranes were blocked with 5% (*w*/*v*) BSA in 0.1% (*v*/*v*) PBS-Tween^®^-20 for 1 h, and then incubated overnight at 4 °C with primary antibodies. After three washes in 0.1% (*v*/*v*) PBS-Tween^®^-20, membranes were incubated for 1 h with specific goat anti-mouse and goat anti-rabbit secondary antibodies (both diluted 1:10,000 in blocking buffer). After three washes in 0.5% (*v*/*v*) PBS-Tween-20, specific protein bands were detected using Clarity Western ECL solution. The Amersham^TM^ 600 Imager (GE Healthcare Life Science, Pittsburgh, PA, USA) was used for detection of chemiluminescent signals, and Quantity One software (4.6.6 version, Bio-Rad) was used to determine the intensity of immunoreactive bands.

### 2.8. Wound Healing Assay

Migration of SH-SY5Y cells was assessed using the wound healing assay. Cells were plated in 6-well plates at a density of 5 × 10^5^ cells/well in complete medium. Upon reaching confluence, the monolayer of cells was cut longitudinally using a 200 μL sterile plastic tip. After three PBS washes to remove non-adherent cells, fresh HI-FBS medium containing TAG or DAG at 5 μM, or vehicle (Ctrl) was added. Cell-free area was observed, and a phase-contrast Nikon TS-100 microscope equipped with a digital acquisition system (Nikon Digital Sight DS Fi-1, Nikon, Minato-ku, Tokyo, Japan) was used to capture images at different times after scratch (0, 3, 7, and 24 h). Cell migration was analysed by measuring and quantifying wound width using the image analysis software ImageJ Version 1.53e (National Institutes of Health, Bethesda, MD, USA).

### 2.9. Statistical Analysis

Statistical analysis was carried out by one-way analysis of variance (ANOVA) followed by Tukey’s HSD test. For Western blot analysis, differences between the housekeeping normalised intensity signals were assessed by Kruskall–Wallis test followed by Conover post-hoc test. Statistical differences were called at *p* < 0.05.

## 3. Results

### 3.1. Isolation and Characterisation of Metabolites from Eurotium chevalieri

**Figure 1:**
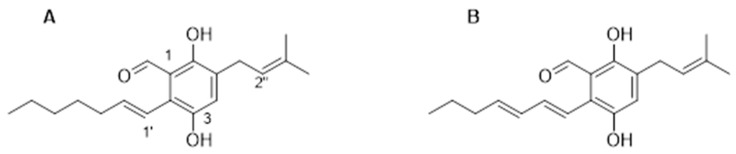
Chemical structures of (**A**) tetrahydroauroglaucin (TAG) and (**B**) dihydroauroglaucin (DAG).

Preliminary LC/MS analysis of the crude intracellular and extracellular extracts from cultures of *Eurotium chevalieri* MUT 2613 revealed an enrichment of prenylated benzaldehyde derivatives in the intracellular extract. Therefore, the intracellular extract was further partitioned in a separation funnel with CHCl_3_:H_2_O (1:1 *v*/*v*), and the organic phase that was collected was dried and purified on a UPLC equipped with an analytical C-18 column, as described in Section 2. Compounds **1**–**6** were identified, and their structures elucidated by HRMS and ^1^HNMR analysis (Figures S2–S7) and by comparison with the literature [4,24]. Based on their abundance in the intracellular extract, TAG and DAG (Figure 1) were evaluated for bioactivity in a cellular in vitro model.

### 3.2. Effects of TAG and DAG on SH-SY5Y Cell Viability

Cell viability was determined by MTT assay. SH-SY5Y cells were treated with different concentrations (2.5, 5, 10, and 20 μM) of TAG and DAG molecules in HI-FBS medium for 24 h.

**Figure 2:**
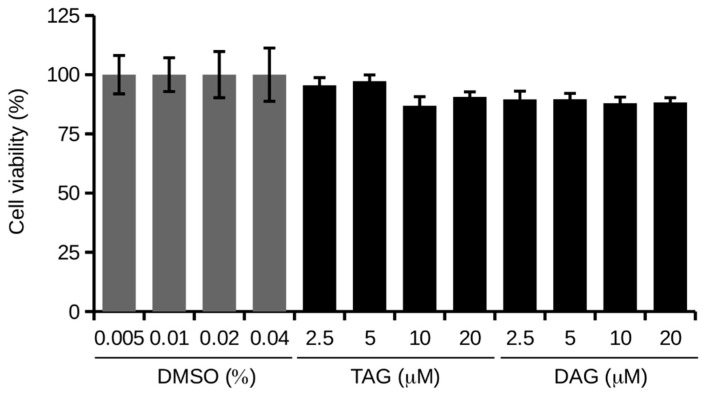
MTT assay on cells treated with TAG and DAG at different concentrations (2.5, 5, 10, and 20 μM) in HI-FBS medium for 24 h. Values are reported as percentages compared to the respective controls, i.e., cells treated with different concentrations of vehicle (DMSO). Data are reported as mean ± SD of three independent experiments. Tukey’s test.

Figure 2 shows that both TAG and DAG only appear to slightly decrease cell viability, but the two molecules have no statistically significant effect on cell viability.

However, the MTT assay measures cellular metabolic activity as an indicator of cell viability. In this work, to confirm the non-toxicity of the molecules TAG and DAG and to rule out cell death after treatments, we verified the effect of TAG and DAG in apoptotic activation by cytofluorimetric analysis.

**Figure 3:**
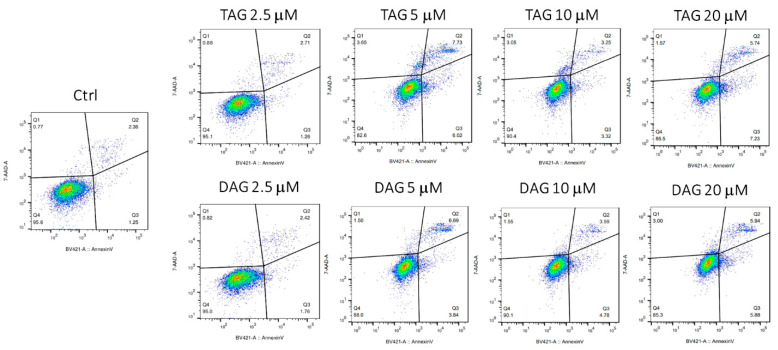
Evaluation of apoptosis in SH-SY5Y cells after the treatment with TAG and DAG molecules at different concentrations (2.5, 5, 10, and 20 μM) for 24 h in HI-FBS medium. The analysis conducted by FACS through Annexin V/7-AAD cell staining is depicted as a typical flow cytometry dot-plot image.

SH-SY5Y cells were treated with different concentrations (2.5, 5, 10, and 20 μM) of TAG and DAG for 24 h in HI-FBS medium and then stained with Annexin V/7-AAD. Vehicle-treated cells were used as controls (Ctrl). Early apoptotic cells (bottom right, Q3) are Annexin V-positive and 7-AAD-negative, late apoptotic cells (top right, Q2) are Annexin V-positive and 7-AAD-positive, and viable cells (bottom left, Q4) are Annexin V-negative and 7-AAD-negative. Under incubation with TAG, SH-SY5Y cells did not show apoptosis by concentrating in the Q4 quadrant, showing a profile comparable to that of control cells. The same occurred in cells treated with DAG at each concentration tested (Figure 3).

These results indicate that TAG and DAG molecules do not induce apoptotic death of SH-SY5Y cells confirming their non-toxicity at the investigated doses.

### 3.3. Effect of TAG and DAG on Autophagy Activation

Many reports have shown that autophagy can play a key role in cancer therapy and that promoting autophagy tends to inhibit the initial step of tumorigenesis [25]. For this reason, the effect of TAG and DAG molecules on autophagy was investigated in this work.

The status of major autophagy proteins was assessed after 24 h of exposure of SH-SY5Y cells to TAG and DAG at the concentrations of 2.5 and 5 μM to verify the potential efficacy of the molecules at the lower doses.

**Figure 4:**
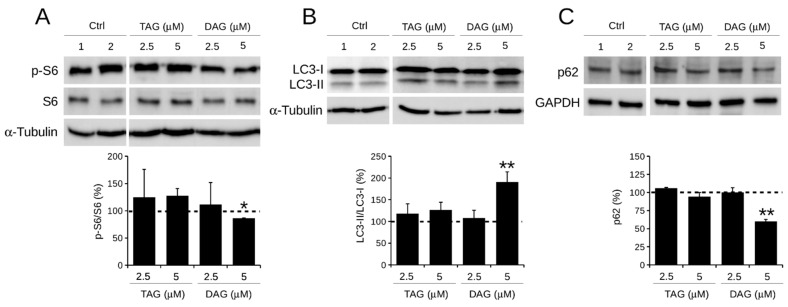
Effect of TAG and DAG on autophagic markers expression in SH-SY5Y cells. Cells were treated with 2.5 or 5 μM of TAG and DAG for 24 h. Control cells (Ctrl) were treated with two doses of DMSO vehicle, i.e., (1) 0.005% DMSO and (2) 0.01% DMSO. Expression levels of protein autophagic markers, i.e., (**A**) p-S6/S6, (**B**) LC3-II/LC3-I, and (**C**) p-62, were detected by Western blot assay. Quantification of signals was determined by densitometry analysis. Error bars represent standard errors. * *p* < 0.05; ** *p* < 0.01 vs. Ctrl (represented by dashed line); Kruskal–Wallis test.

The level of phosphorylation of ribosomal protein S6 (p-S6), a downstream substrate of mTOR—the master negative regulator of autophagy—was analysed. It was found that only cell treatment with DAG (5 μM) caused a reduction in p-S6 by approximately 15% (85 ± 0.6%) compared with control cells (Figure 4A). Although the reduction in p-S6 in DAG-treated cells is small, these data were statistically significant. In contrast, TAG (2.5 and 5 μM) and DAG (2.5 μM) had no effect on p-S6 levels, which remained comparable to those of control cells. Further analysis of the conversion of LC3-I to LC3-II—a recognised marker of autophagosome elongation—found that treatment with 5 μM DAG resulted in an approximately 90% (190 ± 23%) increase in LC3-II/LC3-I, suggesting the presence of autophagosomes in their maturation phase (Figure 4B). Instead, the level of LC3-II/LC3-I in cells treated with TAG (2.5 and 5 μM) and DAG (2.5 μM) was comparable to that of control cells. Finally, when the expression levels of the cargo protein p62—an excellent marker for the final degradative phase of autophagy—were analysed, a clear reduction of approximately 40% (60 ± 2.6%) was observed in cells treated with 5 μM DAG compared with control cells (Figure 4C). This effect was not visible in the other experimental conditions tested.

Overall, these results suggest that only 5 μM DAG activates an autophagic process observable at 24 h of treatment.

### 3.4. Effects of TAG and DAG on SH-SY5Y Cell Migration

**Figure 5:**
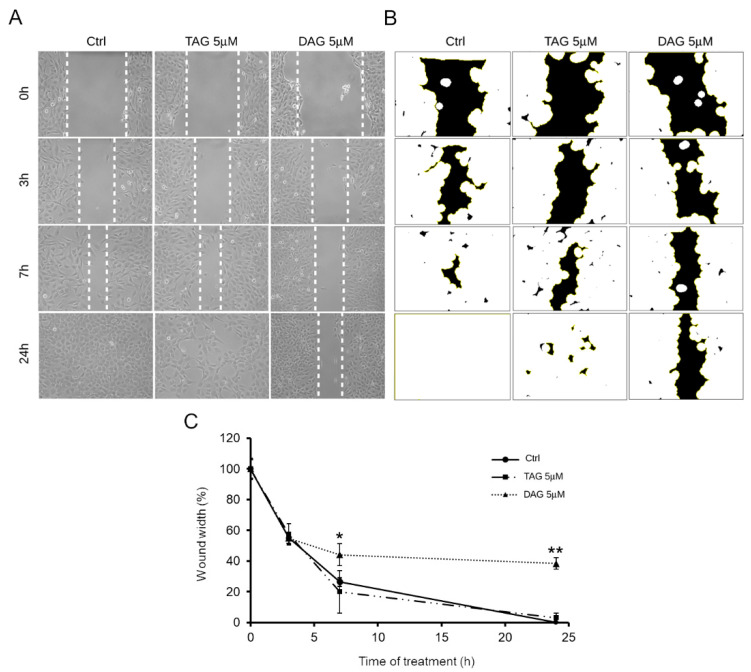
Effect of TAG or DAG on SH-SY5Y cell migration. (**A**) Wound healing assay on SH-SY5Y cells treated with vehicle (Ctrl) or with 5 μM of TAG and DAG. Scratch closure was monitored up to 24 h. Dashed lines mark the edges of the wound area. (**B**) Representative image of wound width (black area) obtained by the image analysis software ImageJ for the quantitative analysis (**C**) Time course analysis of scratch closure in TAG and DAG treated cells or control cells. The wound width for each time point was measured and quantified by using the image analysis software ImageJ. Mean values of wound width at the various time points were reported in percentage terms relative to time 0. Values are represented as means ± SD from three different experiments. Tukey’s test. * *p* < 0.05; ** *p* < 0.01 vs. Ctrl.

Since cell migration is a hallmark of NB cell aggressiveness, this work examined the effect of the two prenylated benzaldehyde derivatives (TAG and DAG) on SH-SY5Y cell migration by wound healing assay. As reported above, 5 μM was the lowest dose at which DAG showed biological activity manifested by an increase in autophagy markers. Therefore, the effect of the two molecules at a concentration of 5 μM on cell migration was tested. Vehicle-treated cells were used as controls. After wounding the cell monolayers, images of the scratched area were captured at different time points (Figure 5A), and the wound width was measured using the image analysis software ImageJ (Figure 5B).

Figure 5C shows that cells treated with TAG (5 μM) exhibited a migration pattern comparable to that of control cells. In contrast, the cells treated with DAG (5 μM) assumed a different migration phenotype than the cells treated with TAG and the control cells as early as after 7 h of treatment. Indeed, at 7 h after the initial scratch, the wound width was approximately 44 ± 7% in DAG-treated cells, whereas at 24 h of treatment after the initial scratch, the wound width was approximately 38 ± 4%. In contrast, wound closure progressed rapidly in cells treated with TAG molecule and reached complete closure at 24 h after treatment as well as in control cells. Taken together, these data suggest that the TAG and DAG, although structurally very similar (Figure 1), do not share the same bioactive properties. Indeed, these results clearly showed that only DAG exerted an inhibitory effect on SH-SY5Y cell migration, underlining a different effectiveness between the two molecules.

## 4. Discussion

Marine organisms are a vast reservoir of bioactive secondary metabolites, a true “blue gold”, as they provide countless molecules with unique chemical diversity useful for the development of new drugs [26]. A key role in the microbial valorisation is played by public culture collections, which safeguard microbial biodiversity and have facilitated access to biological material and associated metadata to boost industrial exploitation of these resources [27].

In the last two decades, tremendous progress has been made in drug design of marine-derived compounds. Several natural products of marine origin are already used in cancer therapy and beyond [28,29] and are part of the global marine clinical pharmaceutical pipeline [30].

Cancer cell migration and invasion are crucial pathological processes that contribute to metastasis and treatment failure in patients with malignancies such as high-risk neuroblastoma (NB) [31,32]. Therefore, preventing cancer metastasis by blocking the highly migratory phenotype of NB cells is undoubtedly a challenge in the fight against cancer progression.

In this study, two prenylated benzaldehyde derivatives, namely tetrahydroauroglaucin (TAG) and dihydroauroglaucin (DAG) were obtained from the intracellular extract of the cultures of the sponge endophyte marine fungus *Eurotium chevalieri* MUT 2613. The two very abundant identified metabolites were investigated as inhibitors of human neuroblastoma SH-SY5Y cell migration. Both molecules were found to have no cellular toxicity and did not activate apoptotic death pathways at the doses tested.

Both TAG and DAG molecules have already been described in the literature to possess antioxidant and radical scavenging activities [9,11,15], antimicrobial, antibiofilm [12], antifouling [3], and anti-inflammatory properties [13], and binding affinity to human opioid or cannabinoid receptors [16].

In addition, data from this wound healing assay demonstrated for the first time that the DAG molecule can inhibit the migration of SH-SY5Y cells. This was not the case with TAG, which conversely showed no bioactive properties. Despite the structural similarity of the two molecules, it is noteworthy that only DAG showed effective abilities to inhibit the highly migratory phenotype of human neuroblastoma SH-SY5Y cells. The presence of an additional double bond in DAG compared with TAG could account for a different mechanism of action of the two molecules and explain the efficacy of one and the inefficacy of the other.

This is not surprising, since the number and the conjugation degree of double bonds in the side chain of the different members of this class of natural products certainly affects the conformational and electronic properties of the whole molecule and thus its interaction with biological targets.

For instance, DAG has been reported to completely inhibit IAV and HSV-1 viral replication, whereas FG was completely ineffective [4]. Similarly, DAG showed more than twofold the antifungal activity against *Candida glabrata* than twice that of TAG and auroglaucin (AG), while FG was again inactive [33].

Similarly, from this study the two prenylated benzaldehyde derivatives (TAG and DAG) also showed different effects on autophagy. Western blot analysis showed that the expression of the phosphorylated form of S6 (p-S6), a downstream substrate of mTOR, was significantly reduced only in DAG-treated cells. At the same time, DAG caused a significant increase in LC3-II/LC3-I levels, an index of autophagosome formation, and a decrease in p62 levels, a marker of the final stage of autophagic degradation.

These results demonstrated the exclusive bioactivity of DAG, and not TAG, in both mTOR-dependent activation of autophagy pathway and inhibition of human neuroblastoma SH-SY5Y cell migration. This finding may support the possible correlation between the effect of DAG on autophagy and inhibition of cell migration. It has been previously described that induction of autophagy in SH-SY5Y cells correlates with inhibition of cell migration [34]. The absence of autophagy activation in TAG-treated cells was also consistent with the ineffectiveness of TAG in inhibiting cell migration. This finding may further support the possible correlation between these two biological processes in human neuroblastoma SH-SY5Y cells.

Although the role of autophagy in cancer remains controversial, autophagy flux could be considered as a new therapeutic target [35]. In this context, our results provided us with a good reason to investigate autophagy/mTOR as a target to reduce neuroblastoma cell migration.

Moreover, since most conventional therapies for NB have a toxic profile [36], it is noteworthy that the naturally derived DAG molecule is effective without signs of cytotoxicity and according to mechanisms that exclude apoptotic death.

In this context, the identification of novel non-toxic migrastatic agents may open new perspectives for optional treatments as single therapy or in combination with lower doses of conventional therapies to limit the high toxicity of anticancer drugs. It is extremely important that migrastatic agents are of low toxicity as long-term administration is required to prevent invasion of cancer cells [19]. The aggressiveness and lethality of high-risk NB is determined by local invasion, but also by the toxicity of anticancer drugs and resistance to conventional therapies. Therefore, with this work we would like to propose DAG as a potentially useful natural marine molecule against the progression of malignant NB cancer. Overall, we believe that our findings provide an additional reason to investigate DAG more thoroughly in further in vitro and in vivo studies.

In conclusion, this work once again highlights the importance of culturomics in the study of microbiomes. Indeed, this approach provides strains that allow for comprehensive characterisation of new species/strains. Making strains available for downstream studies is not trivial and enables the future harnessing of microbial resources in different biotechnological sectors (i.e., unlocking silent or cryptic pathways for secondary metabolites production). In this scenario, a key role is covered by the culture collections: in these facilities fungal and bacterial strains are kept pure and viable, avoiding loss of metabolic potential. They represent a high-quality source for basic research and an indispensable resource for biotechnological purposes.

## Figures and Tables

**Table 1:** Specifications of primary antibodies used in Western blotting experiments.

Primary Antibody	Target	Dilution	Host	Source	Lot
SQTSM1/p62	SQTSM1/p62 protein	1:1000	Rabbit	Abcam	#GR84445-1
LC3	Microtubule-associated protein light chain 3	1:1000	Rabbit	Invitrogen	#UD2753807C
S6	Ribosomial protein S6	1:1000	Rabbit	Cell Signalling	#7
p-S6	Ribosomial protein S6 (Ser235/236)	1:2000	Rabbit	Cell Signalling	#16
GAPDH	Glyceraldehyde 3-phosphate dehydrogenase	1:1000	Mouse	Invitrogen	#UA280593
α-Tubulin	α-Tubulin protein	1:1000	Mouse	Genetex	#43922

## Supplementary Materials

The following are available online at https://www.mdpi.com/article/10.3390/pharmaceutics14030616/s1. Figure S1, ^1^H-NMR spectrum of compound **5** (CDCl3, 400 MHz). Figure S2, HRMS spectrum of compound **1**. Figure S3, HRMS spectrum of compound **2**. Figure S4, HRMS spectrum of compound **3**. Figure S5, HRMS spectrum of compound **4**. Figure S6, HRMS spectrum of compound **5**. Figure S7, HRMS spectrum of compound **6**.
