# Supplementary material for: Dihydroauroglaucin Isolated from the Mediterranean Sponge Grantia compressa Endophyte Marine Fungus Eurotium chevalieri Inhibits Migration of Human Neuroblastoma Cells
Source: Pharmaceutics. 2022 Mar 11;14(3):616. doi: 10.3390/pharmaceutics14030616 (PMC8955805; doi:10.3390/pharmaceutics14030616)
Supplement: Supplementary file 1 [file pharmaceutics-14-00616-s001.zip › pharmaceutics-1574136-supplementary.pdf]

pharmaceutics

Supplementary Materials: Dihydroauroglaucin Isolated from the Mediterranean Sponge *Grantia compressa* Endophyte Marine Fungus *Eurotium chevalieri* Inhibits Migration of Human Neuroblastoma Cells

Marzia Vasarri, Giovanni Andrea Vitale, Giovanna Cristina Varese, Emanuela Barletta, Maria Valeria D'Auria, Donatella de Pascale and Donatella Degl'Innocenti

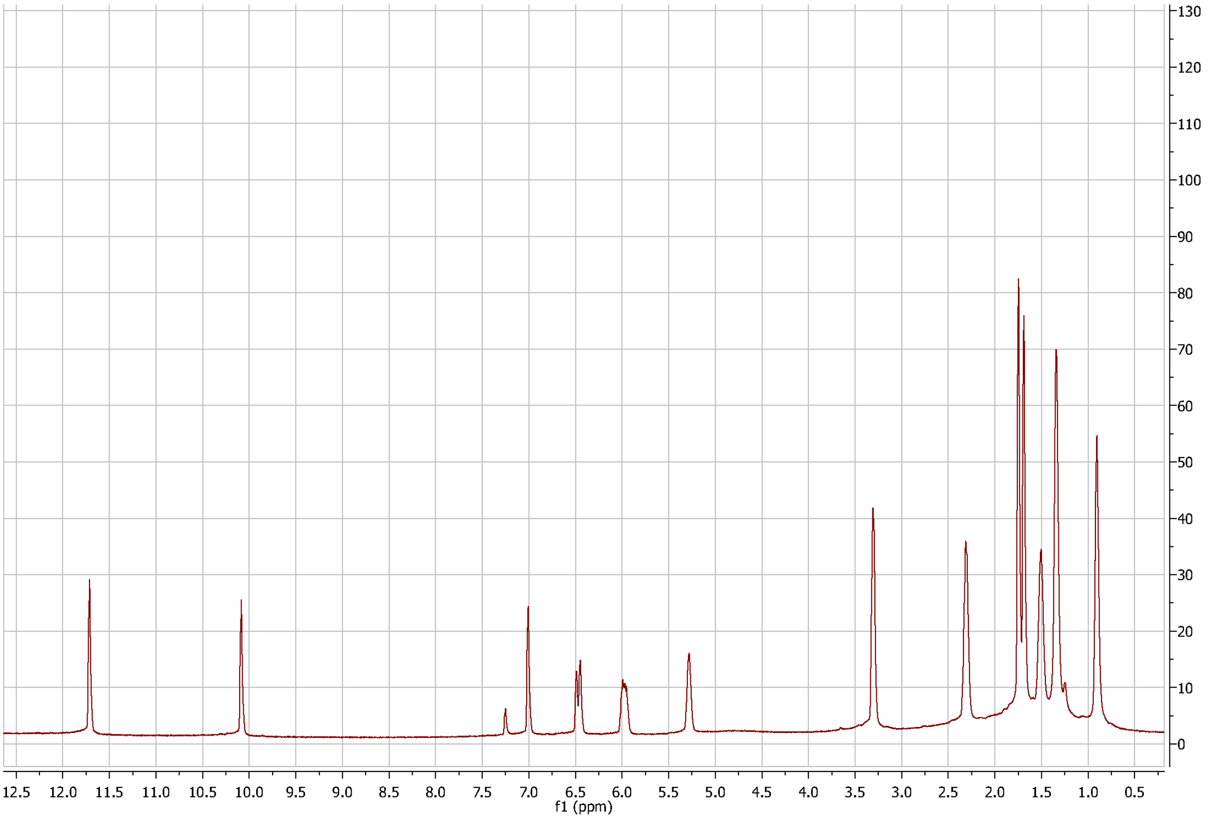

Figure S1. ^{1}H-NMR spectrum of compound **5** (CDCl3, 400 MHz).

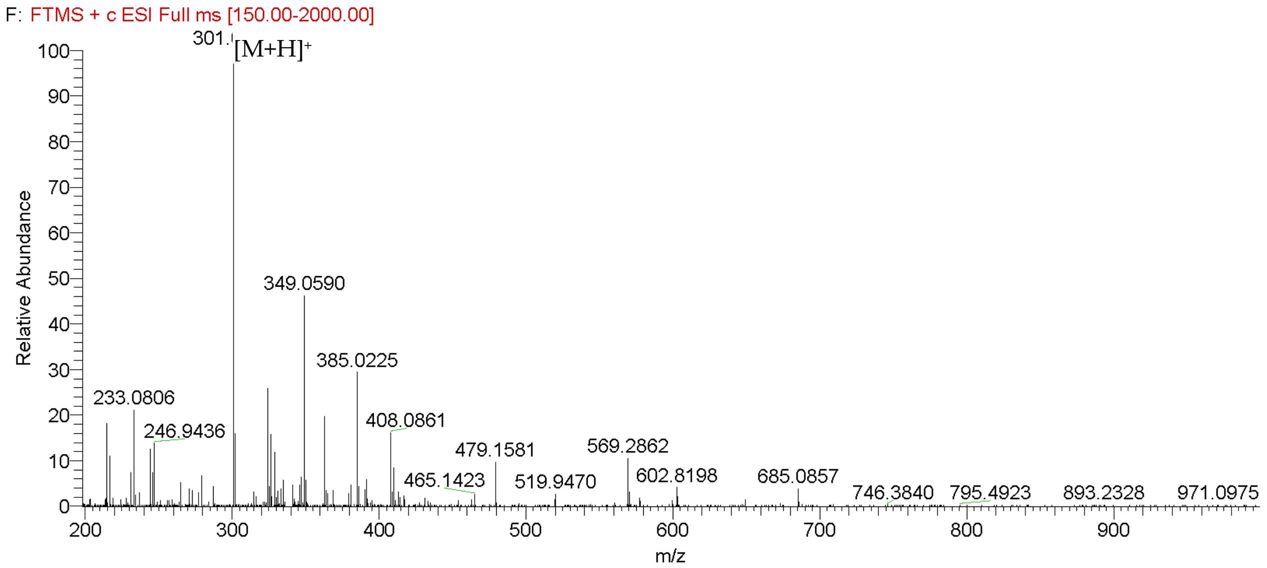

Figure S2. HRMS spectrum of compound **1.**

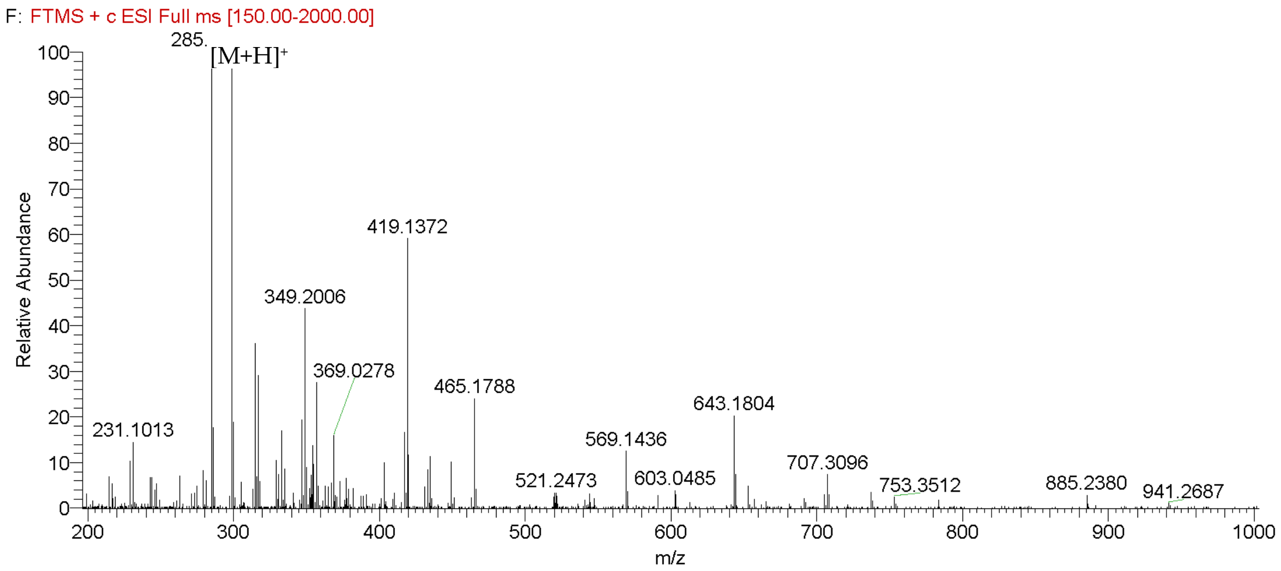

Figure S3. HRMS spectrum of compound **2.**

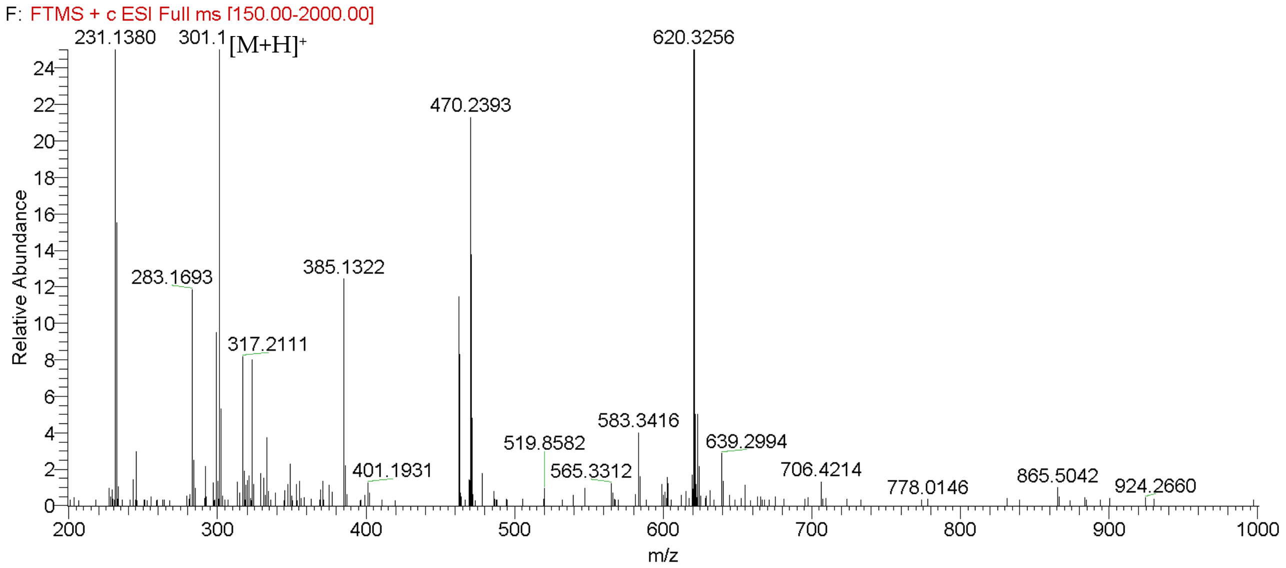

Figure S4. HRMS spectrum of compound **3.**

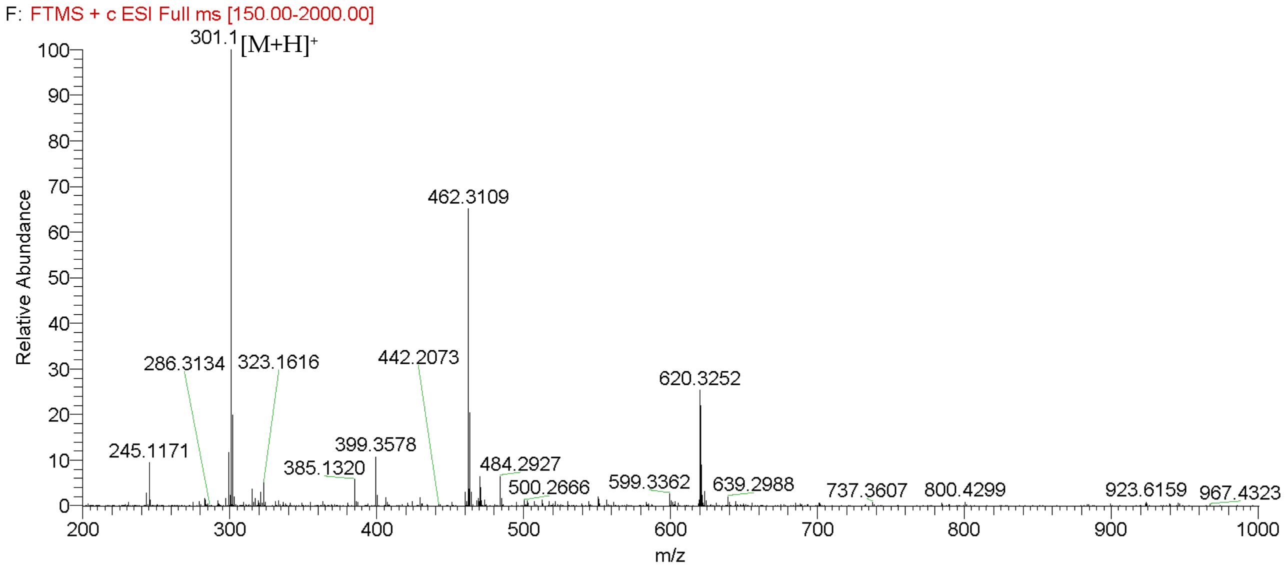

Figure S5. HRMS spectrum of compound **4.**

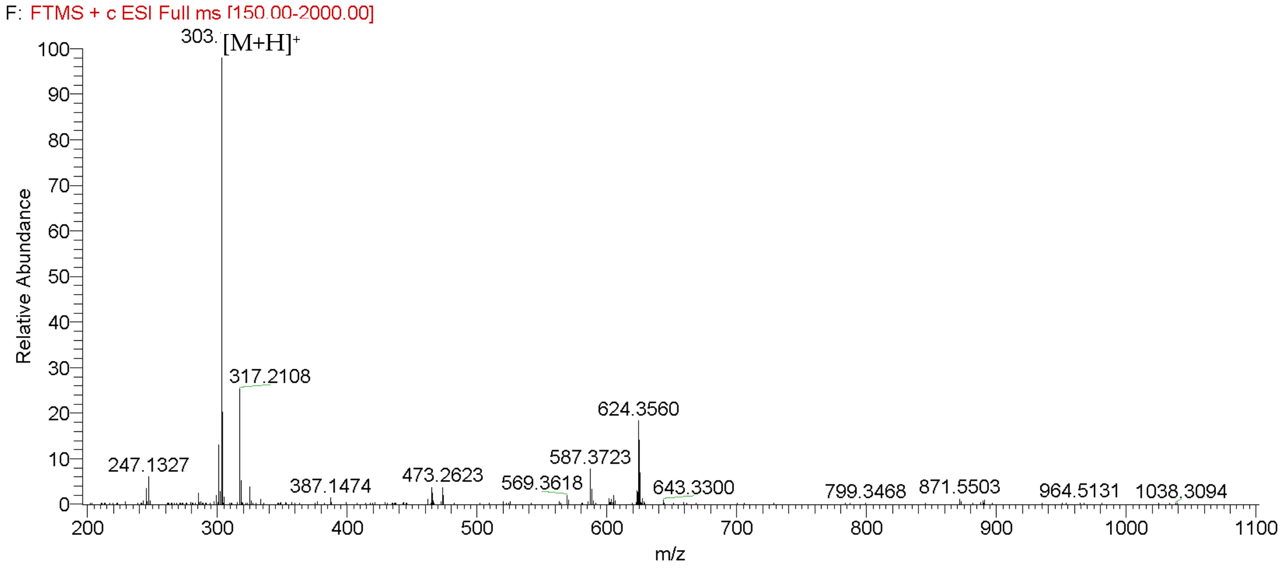

Figure S6. HRMS spectrum of compound **5.**

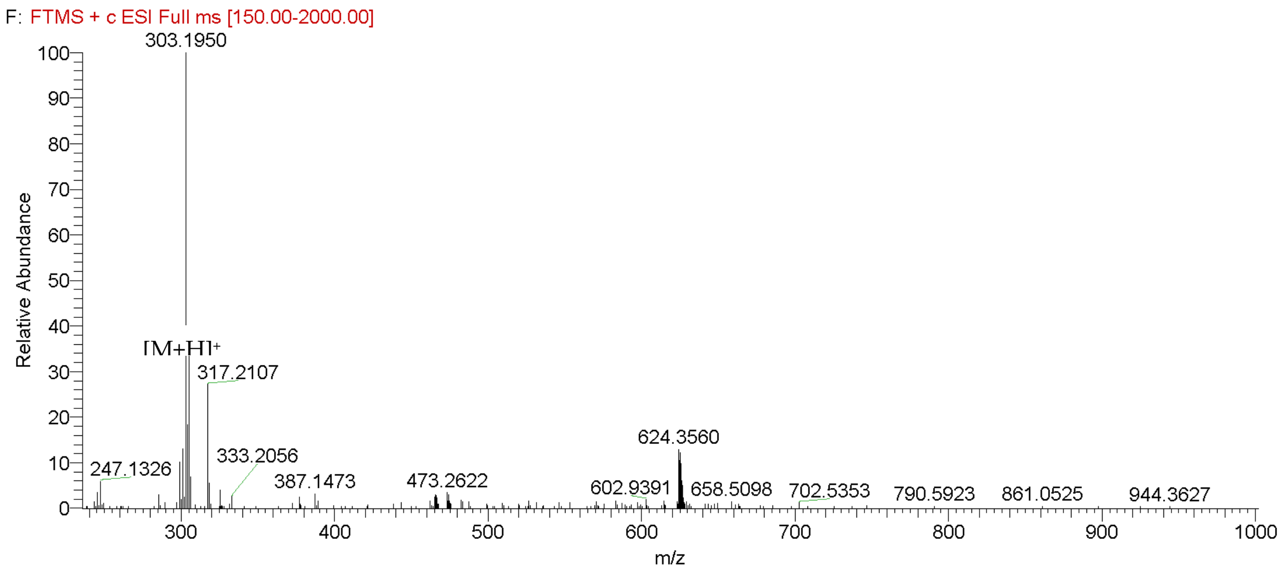

Figure S7. HRMS spectrum of compound **6.**
